# Supplementary material for: Fairness-enhancing classification methods for non-binary sensitive features—How to fairly detect leakages in water distribution systems
Source: PeerJ Comput Sci. 2024 Sep 30;10:e2317. doi: 10.7717/peerj-cs.2317 (PMC11623278; doi:10.7717/peerj-cs.2317)
Supplement: Supplemental Information 1 [file peerj-cs-10-2317-s001.pdf]

A Additional Proofs

A.1 Sigma-Fields

Lemma A.1 (σ-field induced by a set system)**.**
Let Ω be a set and $\mathcal{E} \subset P(\Omega)$ some set system of Ω. Then the set system

$$\sigma(\mathcal{E}) := \bigcap_{\substack{\mathcal{F} \text{ is } \sigma\text{-field on } \Omega, \\ \mathcal{E} \subset \mathcal{F}}} \mathcal{F}$$

defines the smallest σ-field on Ω that holds $\mathcal{E}$, called the σ-field generated by $\mathcal{E}$.

Proof. This is trivial. □

Remark A.2. For the set system $\mathcal{E} \subset P(\Omega)$ of Ω, $\mathcal{E} \subset \sigma(\mathcal{E})$ holds. If the set system $\mathcal{E} \subset P(\Omega)$ is a σ-field on Ω already, $\sigma(\mathcal{E}) = \mathcal{E}$ holds: All σ-fields $\mathcal{F}$ on Ω considered in the intersection satisfy $\mathcal{E} \subset \mathcal{F}$. Therefore, $\mathcal{E} \subset \bigcap_{\mathcal{F} \text{ is } \sigma\text{-field on } \Omega,\ \mathcal{E} \subset \mathcal{F}} \mathcal{F}$ holds. If $\mathcal{E}$ is a σ-field already, $\mathcal{E}$ is one of these σ-fields on Ω that satisfy $\mathcal{E} \subset \mathcal{E}$. Therefore, in this case, $\bigcap_{\mathcal{F} \text{ is } \sigma\text{-field on } \Omega,\ \mathcal{E} \subset \mathcal{F}} \mathcal{F} \subset \mathcal{E}$ also holds.

Remark A.3. If for two set systems $\mathcal{E}_1, \mathcal{E}_2 \subset P(\Omega)$ of Ω, $\mathcal{E}_1 \subset \mathcal{E}_2$ holds, $\sigma(\mathcal{E}_1) \subset \sigma(\mathcal{E}_2)$ holds: All σ-fields that hold $\mathcal{E}_2$ also hold $\mathcal{E}_1$. Therefore, the intersection over all σ-fields that hold $\mathcal{E}_1$ creates an equal or smaller set system compared to the intersection over all σ-fields that hold $\mathcal{E}_2$.

Lemma A.4 (σ-field induced by a function)**.**
Let $X : \Omega \longrightarrow \mathcal{X}$ be a function with σ-field $\mathcal{F}_{\mathcal{X}}$ on $\mathcal{X}$. Then the set system $\sigma(X) := X^{-1}(\mathcal{F}_{\mathcal{X}}) = \{X^{-1}(A) \subset \Omega \mid A \in \mathcal{F}_{\mathcal{X}}\}$ of Ω defines a σ-field on Ω, called the σ-field generated by X.

Proof. (i) $\Omega \in \sigma(X)$: By definition of a σ-field on $\mathcal{X}$, $\mathcal{X} \in \mathcal{F}_{\mathcal{X}}$ holds. Thus, by definition of $\sigma(X)$, $\Omega = X^{-1}(\mathcal{X}) \in \sigma(X)$ holds.

(ii) If $B \in \sigma(X)$, $B^C \in \sigma(X)$, too: If $B \in \sigma(X)$, by definition of $\sigma(X)$, there exists an $A \in F_{\mathcal{X}}$, such that $B = X^{-1}(A)$ holds. By definition of a σ-field, $A^C \in F_{\mathcal{X}}$ holds. Thus, by definition of $\sigma(X)$, $B^C = (X^{-1}(A))^C = X^{-1}(A^C) \in \sigma(X)$ holds.

(iii) If $B_n \in \sigma(X)$ for all $n \in \mathbb{N}$, $\cup_{n\in\mathbb{N}} B_n \in \sigma(X)$, too: If $B_n \in \sigma(X)$, by definition of $\sigma(X)$, there exists an $A_n \in F_{\mathcal{X}}$, such that $B_n = X^{-1}(A_n)$ holds for all $n \in \mathbb{N}$. By definition of a σ-field, $\cup_{n\in\mathbb{N}} A_n \in F_{\mathcal{X}}$ holds. Thus, by definition of $\sigma(X)$, $\cup_{n\in\mathbb{N}} B_n = \cup_{n\in\mathbb{N}} X^{-1}(A_n) = X^{-1}(\cup_{n\in\mathbb{N}} A_n) \in \sigma(X)$ holds. □

Lemma A.5 (σ-field induced by a set system and a function)**.**
Let $X : \Omega \longrightarrow \mathcal{X}$ be a function and $\mathcal{E} \subset P(\mathcal{X})$ some set system of $\mathcal{X}$. Then $X^{-1}(\sigma(\mathcal{E})) = \sigma(X^{-1}(\mathcal{E}))$ holds.

Proof. (i) $\sigma(X^{-1}(\mathcal{E})) \subset X^{-1}(\sigma(\mathcal{E}))$: By remark A.3, $X^{-1}(\mathcal{E}) \subset X^{-1}(\sigma(\mathcal{E}))$ implies $\sigma(X^{-1}(\mathcal{E})) \subset \sigma(X^{-1}(\sigma(\mathcal{E})))$. By lemma A.4 for $\mathcal{F}_{\mathcal{X}} = \sigma(\mathcal{E})$, $X^{-1}(\sigma(\mathcal{E}))$ is a σ-field on Ω. Thus, by remark A.2, $\sigma(X^{-1}(\mathcal{E})) \subset \sigma(X^{-1}(\sigma(\mathcal{E}))) = X^{-1}(\sigma(\mathcal{E}))$ holds.

(ii) $X^{-1}(\sigma(\mathcal{E})) \subset \sigma(X^{-1}(\mathcal{E}))$: By definition of $X^{-1}(\sigma(\mathcal{E}))$, we need to show that for all $A \in \sigma(\mathcal{E})$, $X^{-1}(A) \in \sigma(X^{-1}(\mathcal{E}))$ holds. We do so by using the principle of good sets:

Let $\mathcal{G} := \{A \subset \mathcal{X} \mid X^{-1}(A) \in \sigma(X^{-1}(\mathcal{E}))\}$. The goal is to show that $\sigma(\mathcal{E}) \subset \mathcal{G}$ holds.

(ii.1) $\mathcal{E} \subset \mathcal{G}$: If $A \in \mathcal{E}$, by definition of $X^{-1}(\mathcal{E})$ and remark A.2, $X^{-1}(A) \in X^{-1}(\mathcal{E}) \subset \sigma(X^{-1}(\mathcal{E}))$ holds. Thus, $A \in \mathcal{G}$ holds.

(ii.2) $\mathcal{G}$ is a σ-field on $\mathcal{X}$:
(ii.2.i) $\mathcal{X} \in \mathcal{G}$: By definition of a σ-field on Ω, $X^{-1}(\mathcal{X}) = \Omega \in \sigma(X^{-1}(\mathcal{E}))$ holds. Thus, by definition of $\mathcal{G}$, $\mathcal{X} \in \mathcal{G}$ holds.

(ii.2.ii) If $A \in \mathcal{G}$, $A^C \in \mathcal{G}$, too: If $A \in \mathcal{G}$, by definition of $\mathcal{G}$, $X^{-1}(A) \in \sigma(X^{-1}(\mathcal{E}))$ holds. By definition of a σ-field, $X^{-1}(A^C) = (X^{-1}(A))^C \in \sigma(X^{-1}(\mathcal{E}))$ holds. Thus, by definition of $\mathcal{G}$, $A^C \in \mathcal{G}$ holds.

(ii.2.iii) If $A_n \in \mathcal{G}$ for all $n \in \mathbb{N}$, $\cup_{n\in\mathbb{N}} A_n \in \mathcal{G}$, too: If $A_n \in \mathcal{G}$, by definition of $\mathcal{G}$, $X^{-1}(A_n) \in \sigma(X^{-1}(\mathcal{E}))$ holds for all $n \in \mathbb{N}$. By definition of a σ-field, $X^{-1}(\cup_{n\in\mathbb{N}} A_n) = \cup_{n\in\mathbb{N}} X^{-1}(A_n) \in \sigma(X^{-1}(\mathcal{E}))$ holds. Thus, by definition of $\mathcal{G}$, $\cup_{n\in\mathbb{N}} A_n \in \mathcal{G}$ holds.

Finally, as $\mathcal{E} \subset \mathcal{G}$ and $\mathcal{G}$ is a σ-field on $\mathcal{X}$, by remark A.3 and A.2, $\sigma(\mathcal{E}) \subset \sigma(\mathcal{G}) = \mathcal{G}$ holds, which was the goal to show. □

Remark A.6. If in lemma A.4, the generator of $\mathcal{F}_\mathcal{X}$ is known, i.e., if $\mathcal{F}_\mathcal{X} = \sigma(\mathcal{E}_\mathcal{X})$ holds, using the notation from lemma A.5 with $\mathcal{E} = \mathcal{E}_\mathcal{X}$, we obtain

$$\sigma(X) := X^{-1}(\mathcal{F}_\mathcal{X}) = X^{-1}(\sigma(\mathcal{E}_\mathcal{X})) = \sigma(X^{-1}(\mathcal{E}_\mathcal{X})),$$

i.e., the σ-field generated by the function X equals the σ-field generated by the pre-image of the generator $\mathcal{E}_\mathcal{X}$ of the σ-field $\mathcal{F}_\mathcal{X}$.

A.2 Independence of Two Random Variables

Lemma A.7 (Independence of two random variables, version 1)**.**
X and Y are independent with respect to $\mathbb{P}$ iff

$$\mathbb{P}(X \in A, Y \in B) = \mathbb{P}(X \in A) \cdot \mathbb{P}(Y \in B) \tag{A.11}$$

holds for all $A \in \mathcal{F}_\mathcal{X}, B \in \mathcal{F}_\mathcal{Y}$.

Proof. By definition of $\sigma(X)$ and $\sigma(Y)$ (cf. definition 2.1) and the definition of independence of two families of events (cf. [8]), $\sigma(X)$ and $\sigma(Y)$ are independent iff

$$\mathbb{P}(X^{-1}(A) \cap Y^{-1}(B)) = \mathbb{P}(X^{-1}(A)) \cdot \mathbb{P}(Y^{-1}(B))$$

holds for all $A \in \mathcal{F}_\mathcal{X}, B \in \mathcal{F}_\mathcal{Y}$.[19] Using that

$$\begin{aligned}
&X^{-1}(A) \cap Y^{-1}(B) \\
&= \{\omega \in \Omega \mid X(\omega) \in A\} \cap \{\omega \in \Omega \mid Y(\omega) \in B\} \\
&= \{\omega \in \Omega \mid X(\omega) \in A, Y(\omega) \in B\}
\end{aligned}$$

holds and that $\{X \in A, Y \in B\}$ is just a short form for the latter set, together with analog arguments for $\{X \in A\}$ and $\{Y \in B\}$, we obtain equation (A.11). □

[19] By definition of a random variable, X is $\mathcal{F}$-$\mathcal{F}_\mathcal{X}$- and Y is $\mathcal{F}$-$\mathcal{F}_\mathcal{Y}$-measurable, i.e., for all $A \in \mathcal{F}_\mathcal{X}$, $X^{-1}(A) \in \mathcal{F}$ and for all $B \in \mathcal{F}_\mathcal{Y}$, $Y^{-1}(B) \in \mathcal{F}$ holds. Therefore, the considered probabilities are well defined ($\mathbb{P}$ is a function defined on $\mathcal{F}$).

Lemma A.8 (Independence of two random variables, version 2). *Assume that $\mathcal{F}_\mathcal{X} = \sigma(\mathcal{E}_\mathcal{X})$ and $\mathcal{F}_\mathcal{Y} = \sigma(\mathcal{E}_\mathcal{Y})$ holds and that the set systems $\mathcal{E}_\mathcal{X}$ and $\mathcal{E}_\mathcal{Y}$, also called generators, are $\cap$-stable*[20]*. Then X and Y are independent with respect to $\mathbb{P}$ iff*

$$\mathbb{P}(X \in A, Y \in B) = \mathbb{P}(X \in A) \cdot \mathbb{P}(Y \in B) \tag{A.12}$$

holds for all $A \in \mathcal{E}_\mathcal{X}, B \in \mathcal{E}_\mathcal{Y}$.

Proof. By definition 2.1, X and Y are independent iff $\sigma(X)$ and $\sigma(Y)$ are independent. By definition of these σ-fields (cf. definition 2.1), then X and Y are independent iff $X^{-1}(\sigma(\mathcal{E}_\mathcal{X}))$ and $Y^{-1}(\sigma(\mathcal{E}_\mathcal{Y}))$ are independent. By lemma A.5, $X^{-1}(\sigma(\mathcal{E}_\mathcal{X})) = \sigma(X^{-1}(\mathcal{E}_\mathcal{X}))$ and $Y^{-1}(\sigma(\mathcal{E}_\mathcal{Y})) = \sigma(Y^{-1}(\mathcal{E}_\mathcal{Y}))$ holds. Therefore, X and Y are independent iff $\sigma(X^{-1}(\mathcal{E}_\mathcal{X}))$ and $\sigma(Y^{-1}(\mathcal{E}_\mathcal{Y}))$ are independent.
For the latter case, using that a probability measure $\mathbb{P}$ is uniquely determined by an $\cap$-stable generator of the σ-field it is defined on (cf. [23], lemma 1.42), it suffices to test equation (A.11) on $X^{-1}(\mathcal{E}_\mathcal{X})$ and $Y^{-1}(\mathcal{E}_\mathcal{Y})$, respectively, as these are intersection stable if $\mathcal{E}_\mathcal{X}$ and $\mathcal{E}_\mathcal{Y}$ are. □

Remark A.9 ($\cap$-stable generators are enough). If $\cap$-stable generators $\mathcal{E}_\mathcal{X}$ and $\mathcal{E}_\mathcal{Y}$ of the σ-fields $\mathcal{F}_\mathcal{X}$ and $\mathcal{F}_\mathcal{Y}$, respectively, are known, the following lemmata A.10 and A.12 are replaceable by a version where the σ-fields $\mathcal{F}_\mathcal{X}$ and $\mathcal{F}_\mathcal{Y}$ are replaced by their generators $\mathcal{E}_\mathcal{X}$ and $\mathcal{E}_\mathcal{Y}$, such as we did in lemma A.8 based on lemma A.7.

Lemma A.10 (Independence of two random variables, version 3). *X and Y are independent with respect to $\mathbb{P}$ iff*

$$\mathbb{P}(X \in A) = \mathbb{P}(X \in A \mid Y \in B) \tag{A.13}$$

holds for all $A \in \mathcal{F}_\mathcal{X}$ and $B \in \mathcal{F}_\mathcal{Y}$ for which $\mathbb{P}(Y \in B) > 0$ holds.

Proof. For $A \in \mathcal{F}_\mathcal{X}$ and $B \in \mathcal{F}_\mathcal{Y}$ for which $\mathbb{P}(Y \in B) > 0$ holds, by definition of conditional probabilities (cf. [8]), $\mathbb{P}(X \in A, Y \in B) = \mathbb{P}(X \in A \mid Y \in B) \cdot \mathbb{P}(Y \in B)$ holds. Comparing equation (A.12) and (A.13) yields both implications, noting that for the case $\mathbb{P}(Y \in B) = 0$, equation (A.12) is trivially fulfilled (cf. remark A.11). □

Remark A.11. Conditional probabilities of the kind $\mathbb{P}(X \in A \mid Y \in B)$ are only well-defined for $B \in \mathcal{F}_\mathcal{Y}$ for which $\mathbb{P}(Y \in B) > 0$ holds. However, equation (A.11) is also satisfied for $B \in \mathcal{F}_\mathcal{Y}$ for which $\mathbb{P}(Y \in B) = 0$ holds, because due to rules of (probability) measures, $0 \leq \mathbb{P}(X \in A, Y \in B) \leq \mathbb{P}(Y \in B) = 0$ and therefore, $\mathbb{P}(X \in A, Y \in B) = 0$ holds.

While the results of the previous lemmata are well-known observations, we need a slightly different characterization of independence of random variables than usual to link it to group fairness notions in ML.

Lemma A.12 (Independence of two random variables, version 4). *X and Y are independent with respect to $\mathbb{P}$ iff*

$$\mathbb{P}(X \in A \mid Y \in B_1) = \mathbb{P}(X \in A \mid Y \in B_2) \tag{A.14}$$

holds for all $A \in \mathcal{F}_\mathcal{X}$ and $B_1, B_2 \in \mathcal{F}_\mathcal{Y}$ for which $\mathbb{P}(Y \in B_1), \mathbb{P}(Y \in B_2) > 0$ holds.

[20] A set system $\mathcal{E}$ is called $\cap$-stable iff for any two sets $A_1, A_2 \in \mathcal{E}$, also $A_1 \cap A_2 \in \mathcal{E}$ holds.

Proof. If X and Y are independent with respect to $\mathbb{P}$, equation (A.14) clearly holds for all $A \in \mathcal{F}_\mathcal{X}$ and $B_1, B_2 \in \mathcal{F}_\mathcal{Y}$ for which $\mathbb{P}(Y \in B_1), \mathbb{P}(Y \in B_2) > 0$ holds by lemma A.10.

Vice versa, if equation (A.14) holds for all $A \in \mathcal{F}_\mathcal{X}$ and $B_1, B_2 \in \mathcal{F}_\mathcal{Y}$ for which $\mathbb{P}(Y \in B_1), \mathbb{P}(Y \in B_2) > 0$ holds, we prove that X and Y are independent with respect to $\mathbb{P}$ using lemma A.10 as well. To do so, let $A \in \mathcal{F}_\mathcal{X}$ and $B \in \mathcal{F}_\mathcal{Y}$ for which $\mathbb{P}(Y \in B) > 0$ holds.
Case 1: If $\mathbb{P}(Y \in B) = 1$ holds, by (1) rules of (probability) measures and (2) the definition of conditional probabilities, we obtain

$$0 \overset{(1)}{\leq} \mathbb{P}(X \in A, Y \in B^C) \overset{(1)}{\leq} \mathbb{P}(Y \in B^C) \overset{(1)}{=} 1 - \mathbb{P}(Y \in B) = 0, \text{ and therefore,}$$

$$\begin{aligned}
\mathbb{P}(X \in A) &\overset{(1)}{=} \mathbb{P}(X \in A, Y \in B) + \underbrace{\mathbb{P}(X \in A, Y \in B^C)}_{=0} \\
&\overset{(2)}{=} \mathbb{P}(X \in A \mid Y \in B) \cdot \underbrace{\mathbb{P}(Y \in B)}_{=1} \\
&= \mathbb{P}(X \in A \mid Y \in B).
\end{aligned}$$

Case 2: If $0 < \mathbb{P}(Y \in B) < 1$ holds, by definition of a σ-field, $B^C \in \mathcal{F}_\mathcal{Y}$ and by the assumption of $\mathbb{P}(Y \in B) < 1$, $\mathbb{P}(Y \in B^C) > 0$ holds. Then, the following conditional probabilities are well-defined and we can use equation (A.14): By (1) rules of (probability) measures, (2) the definition of conditional probabilities and (3) this equation (A.14), we obtain

$$\begin{aligned}
\mathbb{P}(X \in A) &\overset{(1)}{=} \mathbb{P}(X \in A, Y \in B) + \mathbb{P}(X \in A, Y \in B^C) \\
&\overset{(2)}{=} \mathbb{P}(X \in A \mid Y \in B) \cdot \mathbb{P}(Y \in B) + \mathbb{P}(X \in A \mid Y \in B^C) \cdot \mathbb{P}(Y \in B^C) \\
&\overset{(3)}{=} \Big(\mathbb{P}(Y \in B) + \mathbb{P}(Y \in B^C)\Big) \cdot \mathbb{P}(X \in A \mid Y \in B) \\
&\overset{(1)}{=} \mathbb{P}(X \in A \mid Y \in B).
\end{aligned}$$

□

B Additional Experimental Results and Analysis

In this section, we present further detailed findings regarding the comparison of all the methods introduced in subsection 4.1.2.

Increasing fairness: In figure B.1, we see the extension of figure 5 for the missing trained ensemble classifiers.
We see that for all fairness-enhancing methods and all leakage sizes, the fairness-enhancing methods on average increase fairness while on average decreasing accuracy by the same or even a smaller amount compared to their corresponding baseline methods. For d = 5, all fairness-enhancing methods allow a large range of fairness improvement at cost of a small range of accuracy, which is only due to the relatively poor accuracy of the leakage detection in this scenario in general. Also for d = 15 and the TFPR- and the ACC-methods with log-barrier function, the range of fairness is larger than the range of accuracy. However, a perfect fairness score of disparate impact being equal to one can not be achieved by these methods. Such result usually comes along with a low accuracy and would increase the

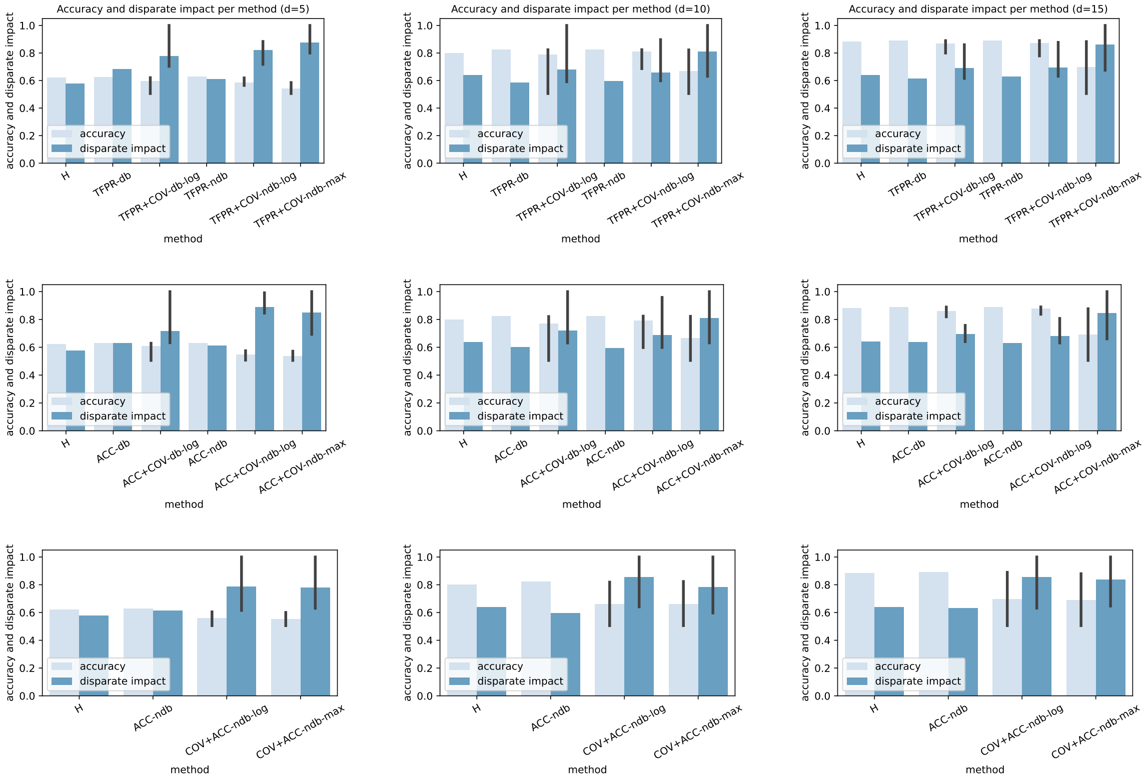

Figure B.1: Accuracy and disparate impact score per method and leakage diameter in the Hanoi-WDS as well as for different hyperparameters c or λ.

accuracy range, as we will see later. For the other scenarios, the ranges of fairness and accuracy are mostly similarly large. Thus, overall, one can say that fairness and overall performance are mutually dependent to about the same extent.
In figure B.2, we see the extension of figure 6 for the missing trained ensemble classifiers. The results do not differ significantly compared to figure 6.

The coherence of fairness and overall performance: In figure B.3, we see the extension of figure 7 for the missing trained ensemble classifier.
Based on our discussions in subsection 3.4, we investigate into the different methods also within the chosen subcategories:
For the TFPR-methods, the methods using the log-barrier yield better results in the sense that their pareto fronts lie above the one using the max-penalty. However, the max-penalty method allows the most fine-grained score combinations, followed by the non-differentiable log-barrier method. As the latter nevertheless allows a disparate impact score larger than 0.8 with a better or similar accuracy score compared to the other TFPR-methods, the TFPR+COV-ndb-log-method is the best performing method among all TFPR-methods.
For the ACC-methods, we observe similar results except that for $d = 10$, the ACC+COV-ndb-log-method even allows the most fine-grained score combinations.
For the COV-methods, the log-barrier method also performs better than the max-penalty method in terms of the position of the pareto-front, but also exhibits some score combinations apart from the pareto-front due to non-convexity of the OP. The non-convexity problem mostly appears for $d = 5$. Nevertheless, as both methods allow fine-grained

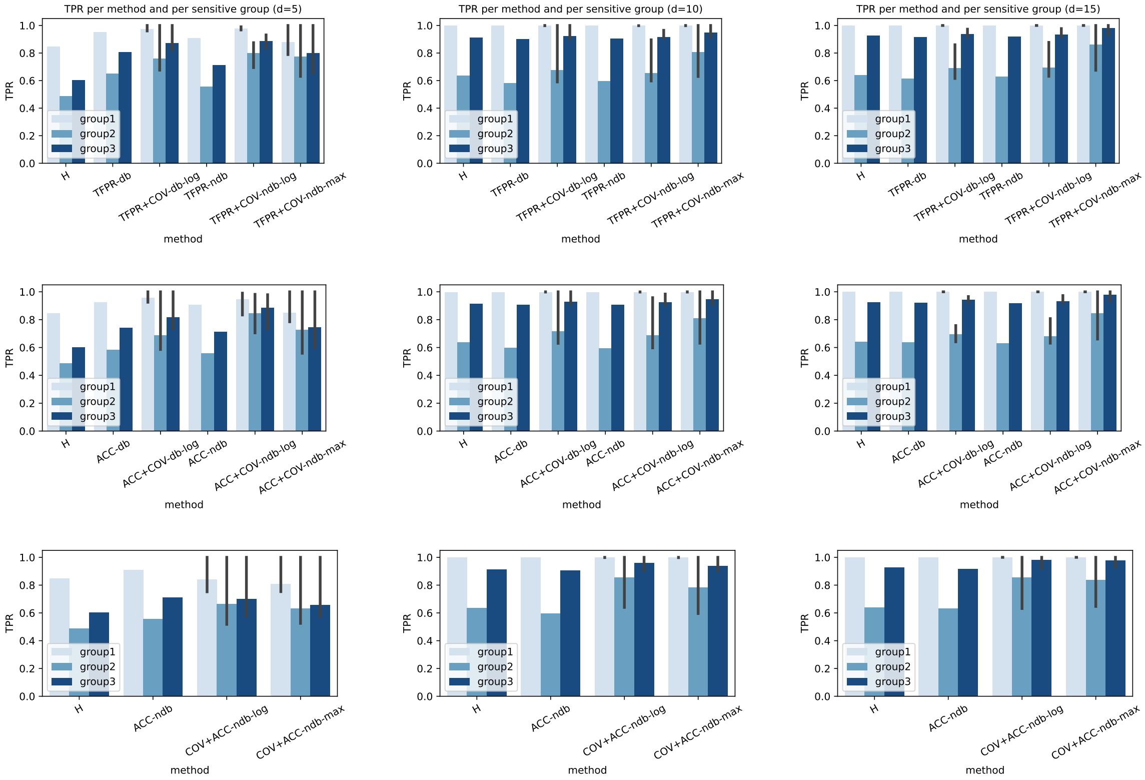

Figure B.2: TPR per method, group and leakage diameter in the Hanoi-WDS as well as for different hyperparameters c or λ.

score combinations, the COV+ACC-ndb-log-method outperforms the COV+ACC-ndb-max-method.

In contrast, for the DI-methods, both log-barrier and max-penalty method provide similarly good pareto-fronts. However, as the max-penalty method allows a little less score combinations apart from the pareto-front and a little more fine-grained score combinations, the DI+ACC-ndb-max-method outperforms the DI+ACC-ndb-log-method.

Also overall, the DI+ACC-ndb-max-method provides the best results: The pareto-front has one of the best shapes (coming closest to the optimal score combination of $(\text{DI}, \text{ACC}) = (1, 1)$) and is finest-grained while having only a few combinations apart from its curve. By that, although for all other subcategories, the log-barrier delivers better results, the best method uses the max-penalty, yielding no clear winner of both of them (cf. paragraph "Algorithmic choices" in subsection 4.1.2). Nevertheless, a large advantage of the max-penalty is the easy choice of the hyperparameter μ: While in this case, μ can be any large number (for us, $\mu = 100$ works), for the log-barrier, the choice of μ requires more finetuning (cf. table 4).

In contrast, rather more obvious is the result that the non-differentiable methods tend to cause better results compared to the differentiable methods, yielding that the error we make when approximating the ensemble classifier is not compensated by the power of the differentiable optimization algorithm (cf. paragraph "Algorithmic choices" in subsection 4.1.2). Another advantage of the non-differentiable methods are that there are less hyperparameters to choose from (cf. table 4).

Overall, the result that the DI+ACC-ndb-max-method provides the best results aligns well with the fact that for this method, the choice of hyperparameters is easiest: While the

hyperparameter μ is easy to choose here as discussed above, also the hyperparameter λ allows a better control of the fairness compared to the hyperparameter c as also elaborated above.

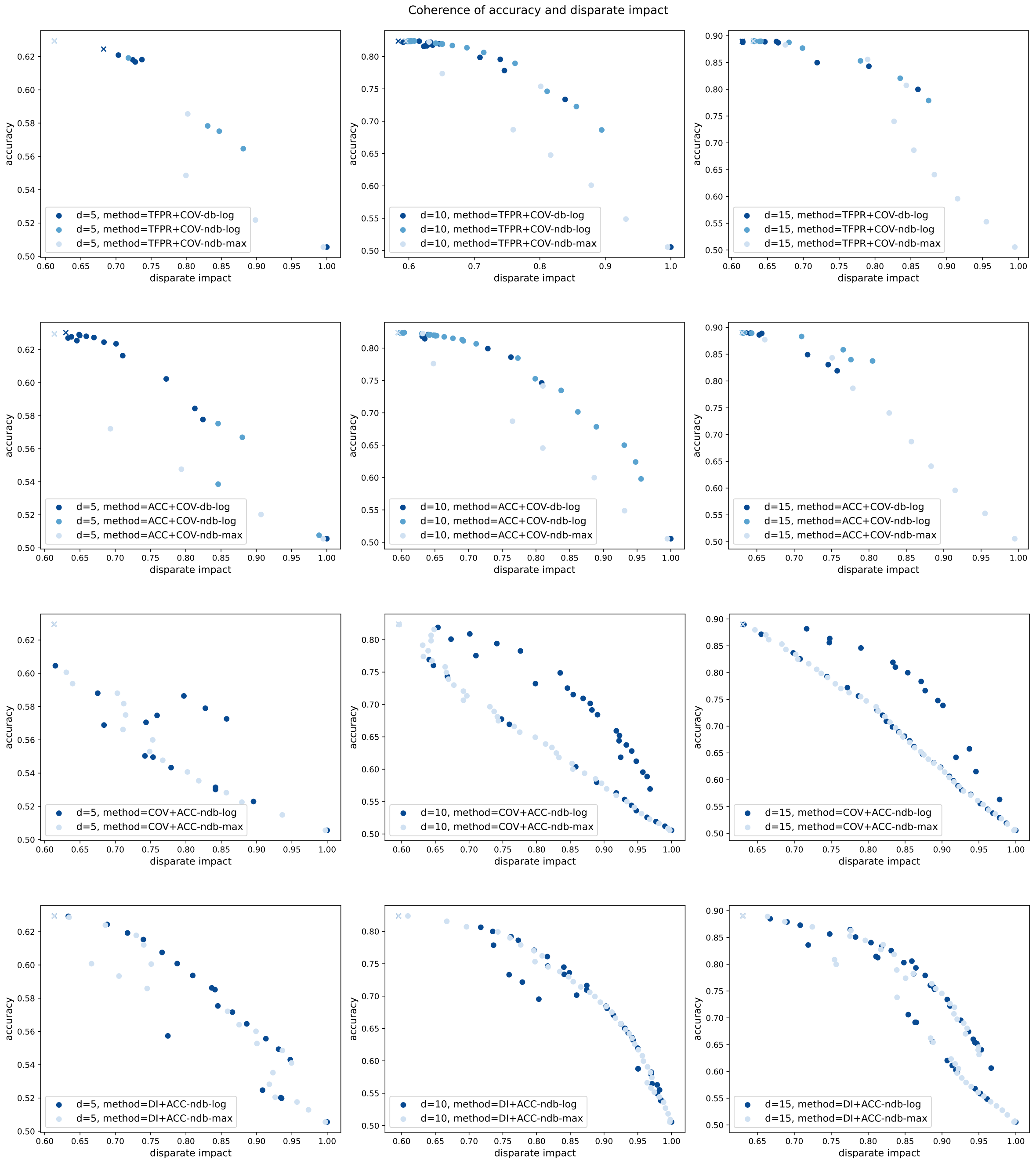

Figure B.3: Coherence of accuracy and disparate impact score for the different fairness-enhancing methods and different leakage sizes in the Hanoi-WDS, based on different hyperparameters c or λ. The cross data points visualize the accuracy and disparate impact score of the corresponding baselines methods (cf. paragraph “Explicit Methods” in subsection 4.1.2 or table 4).
